# Exploring the Association Between Thyroid Function and Oral Microbiome Diversity: An NHANES Analysis

**DOI:** 10.1210/jendso/bvad125

**Published:** 2023-10-05

**Authors:** Liang Zheng, Rui Yang, Ruixia Li, Wanna Chen, Jing Zhang, Weiming Lv, Bo Lin, Jiajun Luo

**Affiliations:** Department of Thyroid Surgery, First Affiliated Hospital of Sun Yat-sen University, Guangzhou, Guangdong 510080, China; Department of Urology, First Affiliated Hospital of Sun Yat-Sen University, Guangzhou, Guangdong 510080, China; Department of Thyroid Surgery, First Affiliated Hospital of Sun Yat-sen University, Guangzhou, Guangdong 510080, China; Department of Thyroid Surgery, First Affiliated Hospital of Sun Yat-sen University, Guangzhou, Guangdong 510080, China; Department of Thyroid Surgery, First Affiliated Hospital of Sun Yat-sen University, Guangzhou, Guangdong 510080, China; Department of Thyroid Surgery, First Affiliated Hospital of Sun Yat-sen University, Guangzhou, Guangdong 510080, China; Department of Thyroid Surgery, First Affiliated Hospital of Sun Yat-sen University, Guangzhou, Guangdong 510080, China; Institute for Population and Precision Health, University of Chicago, Chicago 60637, IL, USA

**Keywords:** oral microbiome, hyperthyroidism, hypothyroidism, thyroid function, thyroid hormones

## Abstract

**Objective:**

To investigate the association between thyroid functions and the oral microbiome diversity.

**Method:**

Data from the US National Health and Nutrition Examination Survey (NHANES; 2009-2012) were analyzed. Thyroid functions were defined using thyroid hormones and related biomarkers. Oral microbiome was measured using the observed number of amplicon sequence variants (ASVs) and the Bray-Curtis dissimilarity. Linear regression was used to estimate the average change (β) and 95% CI for the number of ASVs against thyroid functions, adjusted for sociodemographic variables, health conditions, urinary iodine status, and periodontitis. Non-metric multidimensional scaling (NMDS) was used to analyze the Bray-Curtis dissimilarity.

**Results:**

A total of 2943 participants were analyzed. The observed number of ASVs has a weighted mean of 128.9. Self-reported thyroid disease was associated with reduced number of ASVs (β = −9.2, 95% CI: −17.2, −1.2), if only adjusted for sociodemographic variables and health conditions. In the fully adjusted model, compared to normal thyroid function, both subclinical and clinical hyperthyroidism were associated with reduced number of ASVs (β = −59.6, 95% CI: −73.2, −46.0; β = −28.2, 95% CI: −50.0, −6.5, respectively). Thyroid peroxidase antibody level higher than the reference range was associated with higher observed ASV (β= 9.0, 95% CI: 1.2, 16.9). NMDS analysis suggested significant difference in oral microbiome composition between free triiodothyronine groups (*P* = .002), between free thyroxine groups (*P* = .015), and between thyroglobulin groups (*P* = .035).

**Conclusion:**

Hyperthyroidism was associated with reduced oral microbiome diversity. Free triiodothyronine, free thyroxine, and thyroglobulin levels may alter the oral microbiome composition.

Humans and microbes have coexisted and coevolved in a symbiotic relationship [1]. How human microbiota shape individuals’ health has been a major research focus over the past decade. In addition to the frequently investigated gut microbiome, the oral microbiome is also an essential component of this microorganism community, forming an important microenvironment that contains 500 billion to 1000 billion bacteria [2]. The oral microbiome is located at the beginning of the digestive tract and thus involved in regulating nutrient absorption, substance metabolism, and immune responses [3]. Due to its critical role in human health, the oral microbiome has garnered increasing attention from researchers and clinicians in recent years [4].

Prior studies have documented the close relationship between the oral microbiome and health status. Diseases not limited to oral diseases, but including gastrointestinal, neurological, endocrine, immune, and cardiovascular diseases, can lead to changes in the oral microbiome [5]. Clinical studies have also observed a decrease in the richness and diversity of the oral microbiome during and after radiotherapy [6], as well as considerable changes in the gingival microbiome during pregnancy [7]. Notably, a strong relationship has been established between the oral microbiome and various metabolic diseases or conditions, including diabetes, hyperglycemia, and obesity [8-10]. According to 16S ribosomal RNA sequencing results, diabetes can alter the oral microbiome composition, possibly by the increased expression of interleukin-17 in diabetes patients [11].

Thyroid function is crucial for maintaining normal metabolic function, including energy expenditure, lipid and glucose metabolism, and thermogenesis [12, 13]. Abnormalities in thyroid function, such as hyperthyroidism and hypothyroidism, which are characterized by excessive or insufficient thyroid hormone synthesis, can lead to changes in metabolic rate and body weight as well as alterations in lipid and glucose metabolism [13]. Thyroid hormones also impact the function of other endocrine organs, such as the adrenal glands and pancreas, which further modulate metabolic activities [14, 15]. Recent research has suggested a potential link between thyroid function and the gut microbiome, with alterations in thyroid function potentially affecting the composition of gut microbiota and vice versa [16-20]. For example, alterations in the gut microbiome composition increase the incidence of Hashimoto thyroiditis and Graves’ disease, while changes in the gut microbiome in turn affect thyroid hormone levels by regulating iodine uptake, degradation, and enterohepatic circulation [21].

It is reasonable to hypothesize a reciprocal relationship between thyroid function and the oral microbiome: the oral microbiome participates in the regulation of metabolic processes that involve thyroid function, while altered metabolic processes due to thyroid dysfunction can lead to changes in the oral microbiome. However, information on the relationship between the oral microbiome and thyroid function is limited. Within this context, this study aims to investigate the association between thyroid function and the oral microbiome diversity using the newly released oral microbiome data from the US National Health and Nutrition Examination Survey (NHANES) [22].

## Methods

### Study Population

The NHANES is an ongoing survey, conducted by the National Center for Health Statistics (NCHS) of the Centers for Disease Control and Prevention (CDC), to measure the health and nutrition status of the civilian noninstitutionalized US population ≥2 months of age. The NHANES study protocol is described in detail on the NCHS website (https://www.cdc.gov/nchs/nhanes/index.htm). Informed consent forms are obtained from all NHANES participants.

**Figure 1.**
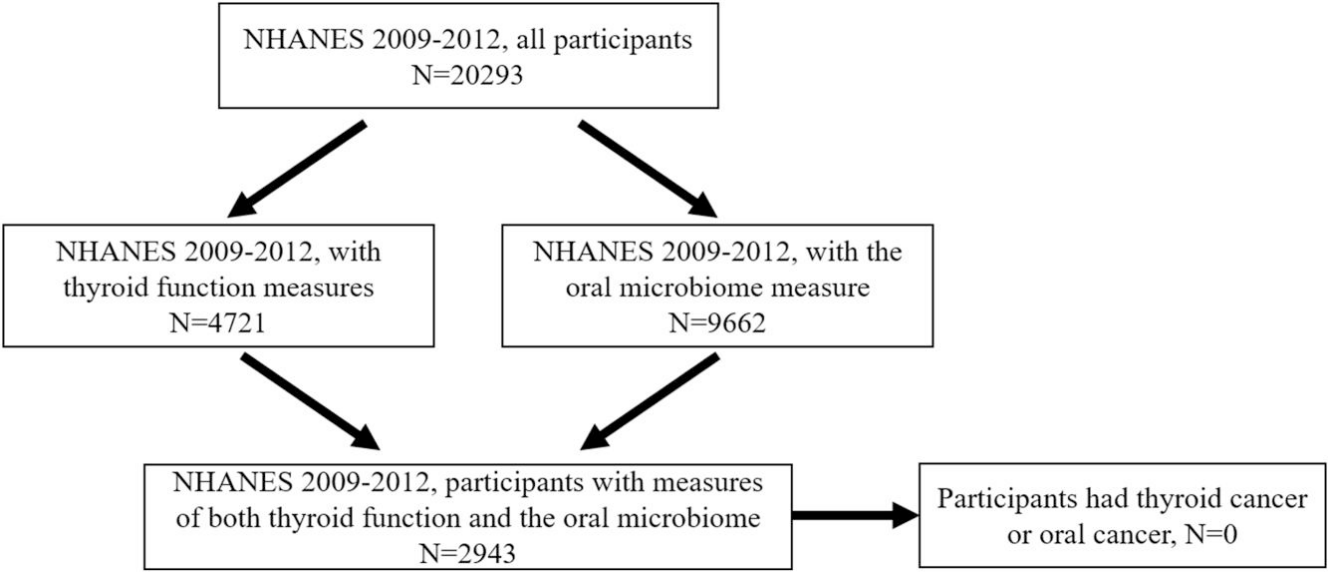
Flowchart for population selection from NHANES 2009-2012 in this study.

The present study used 2 NHANES waves of 2009-2010 and 2011-2012 that include measures of thyroid function and the oral microbiome. The blood samples and oral rinse were collected at the same time when NHANES participants visited the mobile examination centers. We excluded participants who reported a history of thyroid cancer or oral cancer. A flowchart for participant selection can be found in Fig. 1.

### Thyroid Function

Participants self-reported whether they ever had thyroid diseases. Moreover, NHANES contains a battery of tests for the measurement of thyroid function, including total and free thyroxine (TT4 and FT4), total and free triiodothyronine (TT3 and FT3), thyroglobulin (TG), thyroglobulin antibodies (TGAb), thyroid peroxidase antibodies (TPOAb), and thyroid stimulating hormone (TSH). These measurements were conducted at the Collaborative Laboratory Services (Iowa, IA, USA) for NHANES 2009-2010 and 2011-2012, and at the University of Washington (Seattle, WA, USA) for NHANES 2009-2010 using the same methods.

According to the laboratory procedure manuals, the reference ranges for these thyroid measures were: 0.30-5.60 uIU/mL for TSH, 80-180 ng/dL for TT3, 6.10-12.20 ug/dL for TT4, 2.50-3.90 pg/mL for FT3, 0.60-1.60 ng/dL for FT4, 0-4.0 IU/mL for TGAb, 0-35.00 ng/mL for TG, and 0-9.00 IU/mL for TPOAb. We grouped the study population according to these reference ranges. For TSH, TT3, TT4, FT3, and FT4, we defined 3 groups: 1) below the reference range; 2) within the reference range; and 3) above the reference range. For TG, TPOAb, and TGAb, 2 groups were defined: 1) within the reference range; 2) higher than the reference range.

Additionally, we defined subclinical and clinical hypothyroidism and hyperthyroidism according to TSH, FT3, and FT4 for each participant: 1) subclinical hypothyroidism, if TSH was above the reference range, and both FT3 and FT4 were within the reference ranges; 2) subclinical hyperthyroidism, if TSH was below the reference range, and both FT3 and FT4 were within the reference ranges; 3) clinical hypothyroidism, if TSH was above the reference range, and either FT3 or FT4 was lower than the reference range; 4) clinical hyperthyroidism, if TSH was below the reference range, and either FT3 or FT4 was higher than the reference ranges; and 5) normal, if TSH, FT3, and FT4 were all within the reference ranges.

Therefore, the following variables were used to represent thyroid function in this study: self-reported thyroid diseases, subclinical hypothyroidism, subclinical hyperthyroidism, clinical hypothyroidism, clinical hyperthyroidism, TSH group, TT3 group, TT4 group, FT3 group, FT4 group, TGAb group, TG group, and TPOAb group.

### Oral Microbiome Diversity

Oral microbiome testing was performed using oral rinse samples that were originally collected to study the prevalence of oral human papillomavirus in the US population [23]. Details of the procedures for DNA extraction, sequencing, and bioinformatics are available on the NHANES website [22]. Briefly, DNA extracted from these samples were used for bacterial microbiome sequencing. The V4 region of the 16S ribosomal RNA gene was PCR amplified and sequenced. The sequencing data were processed using QIIME and DADA2 software to generate amplicon sequence variant (ASV) tables with taxonomy based on the SILVA version 123 database. A total of 41 378 ASVs were identified.

**Figure 2.**
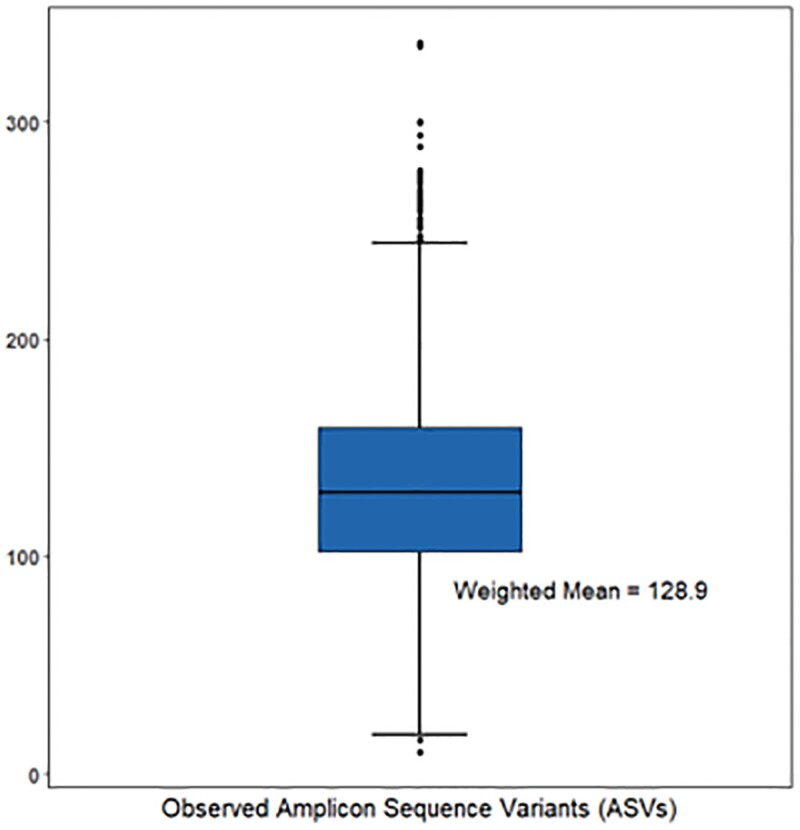
Boxplots of the distribution of 4 alpha-diversity metrics in the study population from NHANES 2009-2012. A) observed amplicon sequence variants (ASVs); B) Faith's phylogenetic diversity; C) the Simpson index; D) the Shannon-Weiner index.

Alpha diversity is a measurement of the microbiome diversity within a single participant, typically representing community richness and community evenness. Four alpha-diversity metrics, including observed ASVs, Faith's Phylogenetic Diversity, the Simpson index, and the Shannon-Weiner Index, were available in NHANES data. The study focused on the observed number of ASVs, a simple measure of alpha diversity that represents the number of unique microbial taxa detected in a sample. This metric is commonly used to compare the richness of different samples or to investigate changes in diversity between exposure groups. The observed number of ASVs appeared to reach saturation by a rarefaction value of 10 000 [22], and this saturated number was analyzed. The distribution of the observed number of ASVs is present in Fig. 2. Other alpha-diversity metrics were also analyzed and their results were presented in the supplemental results [24].

Beta diversity is a measurement of the microbiome diversity between participants, represented by pairwise dissimilarity of participants. In this study, we considered Bray-Curtis dissimilarity [25]. The Bray-Curtis dissimilarity is a popular choice for beta-diversity analysis because it is sensitive to both species’ presence and abundance, and it accounts for differences in the total abundance of species between samples. It is a robust and widely used metric that can be used in various applications, such as comparing microbial community composition across different treatments and assessing community similarity or dissimilarity.

### Periodontitis

Periodontitis was considered in this study, as it is closely related to the oral microbiome diversity. We defined 3 periodontitis groups according to prior publications on oral health in NHANES [26]: a) severe periodontitis, ≥ 2 interproximal sites with attachment loss (AL) ≥ 6 mm (not on the same tooth) and ≥1 interproximal sites with probing depth (PD) ≥ 5 mm; b) moderate/mild periodontitis, ≥ 2 interproximal sites with AL ≥4 mm (not on the same tooth) or ≥2 interproximal sites with PD ≥5 mm (not on the same tooth), or ≥2 interproximal sites with AL ≥3 mm and ≥2 interproximal sites with PD ≥4 mm (not on the same tooth) or one site with PD ≥5 mm; c) and no periodontitis.

### Statistical Analysis

We used linear regression to estimate the average change (β) and corresponding 95% CI for the 4 alpha-diversity metrics against the thyroid function variables. Because current knowledge regarding the association thyroid functions and the oral microbiome is still limited, we consider 3 different models to examine how different confounder sets influence our estimates. Covariates selected for adjustment in these models were considered to have direct effects on both thyroid function and oral health, or to be the proxy variables for the unmeasured confounders. Model 1 adjusted for basic demographic variables race (non-Hispanic White, non-Hispanic Black, Hispanic, other), age (<20, 20-34, 35-49, 50-64, ≥ 65), gender (male, female). No missing values were reported in these covariates. Model 2 additionally adjusted for basic socioeconomic and health-related variables, including education (less than high school, high school or equivalence, some college, college or more), ratio of family income to poverty threshold (<1, 1-2.9, 3-4.9, ≥ 5), body mass index (BMI, < 18.5, 18.5-24.9, 25-29.9, ≥ 30), hypertension (yes, no), type 2 diabetes (yes, no), and self-reported general health conditions (excellent/very good, good, fair/good). These variables are widely considered as confounders in health research. Model 3 further additionally adjusted for variables specifically related to thyroid functions and oral health, including urinary iodine status (low: < 100 µg/L vs normal: ≥ 100 µg/L) and periodontitis (no, moderate/mild, severe). Missing values in the covariates were treated as not missing completely at random for Taylor series variance estimation as presented in the NHANES tutorials.

We used non-metric multidimensional scaling (NMDS), which is commonly regarded as the most robust unconstrained ordination method, to analyze and visualize the overall microbiome compositional difference between participants based on the beta-diversity Bray-Curtis dissimilarity [27]. Analysis of Similarity (ANOSI) was used to test the statistical significance of the difference between thyroid function groups [28].

We conducted a sensitivity analyses to analyze the other 3 alpha-diversity metrics, Faith's Phylogenetic Diversity, the Simpson index, and the Shannon-Weiner Index, in Model 3 to test whether different metrics would alter the results.

All statistical analyses were weighted by appropriate NHANES sample weights and performed using SAS 9.4. The NMDS and ANOSI were conducted using the “vegan” package in R version 4.2.2 [29]. All *P* values were 2-sided, and *P* < .05 was considered statistically significant.

## Results

Table 1 presents the distribution of selected characteristics in the study population. A total of 2943 participants were included. After being weighted by appropriate NHANES sample weights, the study population consisted of 63.9% non-Hispanic White, 12.3% non-Hispanic Black, and 16.0% Hispanic people, which were representative of the actual US civilian noninstitutionalized population in the same time period [30]. The majority of participants were between 20 and 50 years old, and less than 10% had not finished high school. Slightly over 15% of the population had a family income below the poverty threshold. Nearly 80% of the participants self-reported at least good health conditions. The prevalence of hypertension and obesity was 9.5% and 33.1%, respectively, while 18.9% and 5.0% of the participants had moderate/mild and severe periodontitis, respectively. Overall, this study population should be representative of the US general population.

**Table 1.** Unweighted and weighted distributions of sociodemographic and health characteristics in the study population from NHANES 2009-2012

Selected characteristics	Study population (N = 2943*^a^*)	
	Unweighted N (%)	Weighted %*^b^*
**Race**		
Non-Hispanic White	1075 (36.5)	63.9
Non-Hispanic Black	676 (23.0)	12.3
Hispanic	827 (28.1)	16.0
Other	365 (12.4)	7.8
**Age**		
< 20	512 (17.4)	12.0
20-34	769 (26.1)	27.8
35-49	766 (26.0)	29.4
50-64	717 (24.4)	25.8
≥ 65	179 (6.1)	5.0
**Gender**		
Male	1484 (50.4)	49.6
Female	1459 (49.6)	50.4
**Education**		
Less than high school	430 (14.6)	9.7
High school or equivalent	1143 (38.8)	34.1
Some college	800 (27.2)	29.1
College or more	568 (19.3)	26.9
Missing	2 (0.1)	0.1
**Ratio of family income to poverty threshold**		
< 1	709 (24.1)	16.3
1-2.9	1068 (36.3)	31.7
3-4.9	479 (16.3)	23.0
≥ 5	444 (15.1)	22.3
Missing	243 (8.3)	6.8
**Self-report general health condition**		
Excellent/very good	1024 (34.8)	41.9
Good	1145 (38.9)	35.8
Fair/poor	560 (19.0)	15.5
Missing	214 (7.3)	6.8
**Body mass index (BMI)**		
< 18.5	82 (2.8)	2.7
18.5-24.9	938 (31.9)	32.7
25-29.9	887 (30.1)	30.6
≥ 30	1010 (34.3)	33.1
Missing	26 (0.9)	0.9
**Hypertension**		
No	2517 (85.5)	87.6
Yes	323 (11.0)	9.5
Missing	103 (3.5)	3.0
**Type 2 diabetes**		
No	2620 (89.0)	90.7
Yes	322 (10.9)	9.3
Missing	1 (0)	0
**Urinary iodine status**		
Normal	1832 (62.3)	62.8
Low	1086 (36.9)	36.5
Missing	25 (0.8)	0.7
**Periodontitis**		
No	941 (32.0)	39.1
Moderate/mild	595 (20.2)	18.9
Severe	214 (7.3)	5.0
Missing	1193 (40.5)	36.9
**Self-report thyroid disease history**		
No	2233 (75.9)	79.7
Yes	196 (6.7)	8.3
Missing	514 (17.5)	12.0
**Thyroid disorders**		
Normal	2586 (87.9)	87.3
Subclinical hypothyroidism	39 (1.3)	1.9
Subclinical hyperthyroidism	1 (0.0)	0.0
Clinical hypothyroidism	13 (0.4)	0.4
Clinical hyperthyroidism	14 (0.5)	0.4
Missing	290 (9.9)	10.0
**Thyroid stimulating hormone group**		
Above reference range (<0.3 uIU/mL)	53 (1.8)	2.4
Within reference range (0.3-1.18 uIU/mL)	2840 (96.5)	95.7
Below reference range (>5.60 uIU/mL)	48 (1.6)	1.8
Missing	2 (0.1)	0.1
**Total triiodothyronine group**		
Above reference range (<80 ng/dL)	64 (2.2)	1.9
Lower tertile within reference range (80-180 ng/dL)	2808 (95.4)	96.0
Below reference range (>180 ng/dL)	68 (2.3)	2.0
Missing	3 (0.1)	0.1
**Total thyroxine group**		
Above reference range (<6.10 ug/dL)	48 (1.6)	1.4
Within reference range (6.10-12.20 ug/dL)	2648 (90.0)	89.3
Below reference range (>12.20 ug/dL)	218 (7.4)	8.3
Missing	29 (1.0)	1.0
**Free triiodothyronine**		
Above reference range (<2.50 pg/mL)	160 (5.4)	5.1
Within reference range (2.50-3.90 pg/mL)	2724 (92.6)	92.9
Below reference range (>3.90 pg/mL)	51 (1.7)	1.8
Missing	8 (0.3)	0.2
**Free thyroxine**		
Above reference range (<0.60 ng/dL)	10 (0.3)	0.3
Within reference range (0.60-1.60 ng/dL)	2859 (97.2)	97.1
Below reference range (>1.60 ng/dL)	72 (2.5)	2.5
Missing	2 (0.1)	0.1
**Thyroglobulin antibodies**		
Within reference range (≤4.0 IU/mL)	2721 (92.5)	91.5
Above reference range (>4.0 IU/mL)	197 (6.7)	7.6
Missing	25 (0.9)	0.9
**Thyroglobulin**		
Within reference range (≤35.00 ng/mL)	2768 (94.1)	93.7
Above reference range (>35.00 ng/mL)	170 (5.8)	6.1
Missing	5 (0.2)	0.1
**Thyroid peroxidase antibodies**		
Lower tertile within reference range (≤9.00 IU/mL)	2638 (89.6)	87.5
Above reference range (>9.00 IU/mL)	273 (9.3)	11.5
Missing	32 (1.1)	1.0

a Unweighted number of study population.

b Weighted by NHANES sample weight.

In the weighted population, less than 10% self-reported a history of thyroid diseases. According to the thyroid hormone measures, 87.3% of participants had normal thyroid function. Subclinical hypothyroidism had the highest prevalence, of 1.9%, while both clinical hypothyroidism and clinical hyperthyroidism had a prevalence of 0.4%.

Figure 2 shows the distribution of the observed number of ASVs in this study. The weighted mean number was 128.9 in this study. The associations between thyroid function and the observed number of ASVs can be found in Table 2. We observed significant association between self-reported thyroid disorders and ASVs in Model 1 (β = −8.6, 95% CI: −15.9, −1.3) and Model 2 (β = −9.2, 95% CI: −17.2, −1.2); however, when additionally adjusted for urinary iodine status and periodontitis, the association became borderline significant in Model 3 (β = −8.2, 95% CI: −17.2, 0.8). Subclinical hyperthyroidism was significantly associated with reduced diversity in all models, and the magnitudes of the association were strengthened when additionally adjusted for more covariates, from −48.4 (95% CI: −52.8, −44.0) in Model 1, to −53.3 (95% CI: −63.8, −42.8) in Model 2, and eventually to −59.6 (95% CI: −73.2, −46.0) in Model 3. Clinical hyperthyroidism was significantly associated with reduced microbiome diversity in Model 2 (β = −28.7, 95% CI: −56.8, −0.6). This association became more robust when additionally adjusted for urinary iodine and periodontitis in Model 3 (β = −28.2, 95% CI: −50.0, −6.5). Notably, the association of clinical hypothyroidism with the microbiome diversity was positive in Model 1 (β = 8.0, 95% CI: −11.3, 27.2), Model 2 (β = 10.2, 95% CI: −10.9, 31.2), and Model 3 (β = 11.3, 95% CI: −9.4, 32.0); however, these associations were not significant.

**Table 2.** Association between thyroid function and the observed number of amplicon sequence variants (ASVs) in the linear regression model

	Linear coefficient for the observed number of ASVs [β (95% CI)]		
	Model 1	Model 2	Model 3
**Self-report thyroid disease**			
No	Ref	Ref	Ref
Yes	−8.6 (−15.9, −1.3)	−9.2 (−17.2, −1.2)	−8.2 (−17.2, .8)
**Thyroid disorders**			
Normal	Ref	Ref	Ref
Subclinical hypothyroidism	−5.1 (−16.6, 6.4)	3.8 (−7.8, 15.4)	3.8 (−13.7, 21.3)
Subclinical hyperthyroidism	−48.4 (−52.8, −44.0)	−53.3 (−63.8, −42.8)	−59.6 (−73.2, −46.0)
Clinical hypothyroidism	8.0 (−11.3, 27.2)	10.2 (−10.9, 31.2)	11.3 (−9.4, 32.0)
Clinical hyperthyroidism	−27.7 (−59.5, 4.1)	−28.7 (−56.8, −0.6)	−28.2 (−50.0, −6.5)
**Thyroid stimulating hormone group**			
Above the reference range	−3.2 (−13.2, 6.9)	4.1 (−6.4, 14.5)	1.4 (−13.4, 16.3)
Within the reference range	Ref	Ref	Ref
Below the reference range	−11.7 (−29.3, 5.8)	−13.9 (−30.8, 3.0)	−11.7 (−34.0, 10.6)
**Total triiodothyronine group**			
Above the reference range	2.6 (−8.1, 13.2)	0.3 (−10.9, 11.5)	−5.4 (−19.9, 9.2)
Within the reference range	Ref	Ref	Ref
Below the reference range	−0.5 (−10.2, 9.2)	−0.9 (−10.1, 8.4)	−1.8 (−11.6, 8.0)
**Total thyroxine group**			
Above the reference range	−4.9 (−17.6, 7.9)	−2.2 (−15.7, 11.2)	−2.7 (−16.1, 10.7)
Within the reference range	Ref	Ref	Ref
Below the reference range	2.3 (−4.0, 8.7)	3.3 (−3.6, 10.2)	−0.1 (−7.7, 7.4)
**Free triiodothyronine**			
Above the reference range	−1.4 (−9.0, 6.1)	1.1 (−6.7, 9.0)	8.3 (−7.6, 24.3)
Within the reference range	Ref	Ref	Ref
Below the reference range	11.1 (−2.9, 25.0)	10.2 (−1.7, 22.0)	−.5 (−10.8, 9.8)
**Free thyroxine**			
Above the reference range	−24.7 (−63.5, 14.1)	−27.7 (−63.9, 8.5)	−23.9 (−50.8, 2.9)
Within the reference range	Ref	Ref	Ref
Below the reference range	−1.5 (−16.4, 13.3)	0.1 (−16.2, 16.4)	−10.5 (−27.5, 6.5)
**Thyroglobulin antibodies**			
Within the reference range	Ref	Ref	Ref
Above the reference range	−3.4 (−13.5, 6.8)	2.7 (−7.4, 12.9)	2.9 (−9.6, 15.5)
**Thyroglobulin**			
Within the reference range	Ref	Ref	Ref
Above the reference range	5.2 (−1.4, 11.7)	1.4 (−6.0, 8.8)	1.9 (−7.9, 11.8)
**Thyroid peroxidase antibodies**			
Within the reference range	Ref	Ref	Ref
Above the reference range	3.5 (−2.8, 9.8)	7.5 (0.6, 14.4)	9.0 (1.2, 16.9)

Model 1 adjusted for race (non-Hispanic White, non-Hispanic Black, Hispanic, other), age (<20, 20-34, 35-49, 50-64, ≥ 65), and gender (male, female).

Model 2 adjusted for education (less than high school, high school or equivalence, some college, college or more), ratio of family income to poverty threshold (<1, 1-2.9, 3-4.9, ≥ 5), body mass index (BMI, < 18.5, 18.5-24.9, 25-29.9, ≥ 30), hypertension (yes, no), type 2 diabetes (yes, no), and self-reported general health conditions (excellent/very good, good, fair/good), in addition to covariates in Model 1.

Model 3 adjusted for urinary iodine status (normal, low) and periodontitis (no, moderate/mild, severe) in addition to covariates in Model 2.

No significant association was observed with most thyroid hormone and biomarker groups, including TSH, TT3, TT4, FT3, FT4, TGAb, and TG; however, a significant positive association with microbiome diversity was observed for high TPOAb. TPOAb was not significantly associated with microbiome diversity in Model 1 (β = 3.5, 95% CI: −2.8, 9.8), but this association became significant and stronger in Model 2 (β = 7.5, 95% CI: .6, 14.4), and further strengthened in Model 3 (β = 9.0, 95% CI: 1.2, 16.9).

When using other alpha-diversity metrics other than the observed number of ASVs (distributions in Supplemental Figure S1 [24]), subclinical hyperthyroidism was still significantly associated with reduced oral microbiome diversity (Supplemental Tables S1-S3 [24]). However, the association of clinical hyperthyroidism was borderline to being considered significant. High TPOAb group was also borderline significantly associated with higher Faith's phylogenetic diversity in Model 3 (β = −0.7, 95% CI: 0, 1.5), but was not with the other 2 indices.

**Figure 3.**
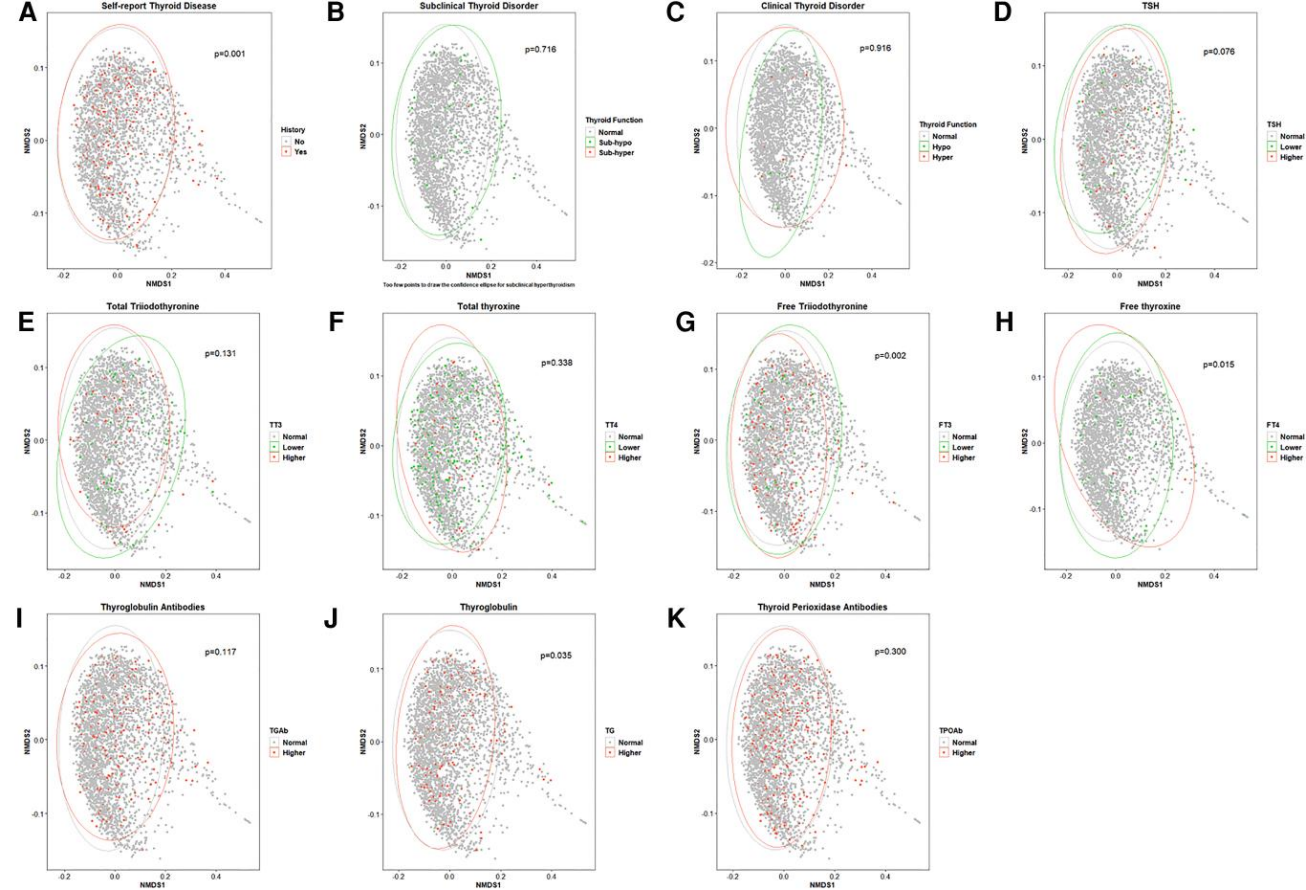
The Non-metric Multidimensional Scaling (NMDS) plot that represents the oral microbiome composition of each participant in NHANES 2009-2012, grouped by thyroid function variables. The shorter the distance between the dots, the more similar the bacterial compositions were between the patients. The ellipse represents the 95% confidence area for each group. A) self-report thyroid disease history; B) subclinical thyroid disorder; C) clinical thyroid disorder; D) thyroid stimulating hormone group; E) free triiodothyronine; F) free thyroxine; G) total triiodothyronine; H) total thyroxine; I) thyroglobulin; J) thyroglobulin antibodies; K) thyroid peroxidase antibodies.

Analysis of beta diversity shows the difference in the oral microbiome composition across different thyroid function groups (Fig. 3). Self-reported thyroid disease was also significantly associated with different microbiome composition (*P* = .001), even though the difference was small in the figure. Although significant associations were observed for subclinical and clinical hyperthyroidism with the observed number of ASVs, no significant difference in the microbiome composition was observed when comparing subclinical or clinical hyperthyroidism with normal thyroid function groups (*P* = .716 and .916, respectively). A significant difference in the microbiome composition was observed across FT3, FT4, and TG groups (*P* = .002, .015, and .035, respectively). For FT3, the microbiome distribution of higher group was narrower and appeared to be a subset of the lower group. For FT4, the non-overlapped area between higher and lower groups was non-ignorable. For TG, although a significant difference was noted, the distribution was quite similar between the normal and higher groups. Borderline significant difference was also observed for TSH (*P* = .076), for which the lower group demonstrated a narrower distribution of microbiome composition compared to the higher group.

## Discussion

This is a large-scale study to investigate the association between thyroid functions and oral microbiome diversity. We found that both subclinical and clinical hyperthyroidism was associated with reduced oral microbiome diversity, and high TPOAb was associated with higher microbiome diversity. In terms of the microbiome composition between participants, findings from this study suggested difference in the composition across FT3, FT4, and TG groups. Overall, the study provides evidence for an association between the oral microbiome and thyroid function, especially hyperthyroidism.

Microbial diversity is often closely related to the health status of the host. Generally, in the absence of oral diseases, healthier hosts have a higher oral microbiome diversity. In this study, we observed both positive and inverse associations with different thyroid functions. To our knowledge, only one prior study linked higher TSH with increased oral microbiome diversity [31]. A similar but nonsignificant positive association was also observed in our study. Although evidence regarding the association between the oral microbiome and thyroid function is sparse, research on the gut microbiome may provide some clues, because these 2 microbial communities are related to each other [32, 33]. Research has shown that oral interventions, regardless of beneficial (periodontal treatment) or detrimental (oral ingestion of *Porphyromonas gingivalis*), demonstrated the capabilities to alter the downstream gut microbiome [32, 33].

In a pilot clinical research, researchers found that the levels of *Bifidobacterium* and *Lactobacillus* in the gut microbiome were significantly lower in 14 patients with hyperthyroidism than in 7 healthy controls [34]. Patients with Graves’ disease, the most common form of hyperthyroidism, have been shown to have significantly lower alpha diversity and abundance of gut microbiota compared to healthy controls [35]. Additionally, the study observed that the microbiome diversity metrics improved after treatment and restoration of thyroid function among these patients, suggesting that the decrease in the microbiome diversity may be closely related to the progression of the disease [35]. These findings were consistent with the association observed in this study between hyperthyroidism and reduced alpha-diversity metrics.

However, the evidence for hypothyroidism and human microbiome is more mixed. One study suggests that the primary hypothyroidism is related to a significant reduction in the number of strains producing propionic and butyric acid in the gut of patients [36]. On the other hand, investigations of the gut microbiome composition observed both dysbiosis and bacterial overgrowth in patients with hypothyroidism [37, 38]. Moreover, research suggests that TPOAbs play a role in hypothyroidism. In a 20-year follow-up study in the UK, patients with elevated TPOAb levels had a higher progression rate to overt hypothyroidism compared to TPOAb-negative patients [39]. Interestingly, there is evidence to support an association between TPOAb level and the enrichment of oral microbiota in patients with thyroid nodules [40]. Despite the oral and gut microbiome communities having distinct taxonomic features at the phylum and genus levels [41-43], prior findings regarding the gut microbiome still support observations about the oral microbiome in this study.

One interesting observation is that the significant association was observed with clinical or subclinical hyperthyroidism, but not with individual triiodothyronine or thyroxine levels. Prior research demonstrated that clinical and subclinical hyperthyroidism are associated with adverse health outcomes including heart failure, osteoporosis, and dementia, particularly among populations aged 65 or above [44]. In comparison, changes in individual triiodothyronine or thyroxine levels may not necessarily have clinical significance [44], which may explain why the significant associations was only observed with clinical or subclinical hyperthyroidism. However, triiodothyronine or thyroxine still demonstrated consistent, though nonsignificant, inverse associations with the oral microbiome diversity in this study.

Moreover, experiment studies suggest that human microbiome is widely involved in triiodothyronine and thyroxine metabolism [21, 45, 46]. Triiodothyronine can be conjugated and excreted as sulfate glucuronide derivatives (T3S). T3S is considered a reservoir of iodinated thyroglobulin, especially in fetal tissues, and excreted T3S can be restored via the action of intestinal bacterial sulfatases [46]. Thyroxine can be conjugated to glucuronic acid (T4G), which would be detoxified by the human microbiome and subsequently reabsorbed by the host. Alternatively, T4G may bind to the microbiome for storage and later release. Unconjugated T4 can also bind to bacteria in the intestines [21, 45]. The close relationship of triiodothyronine and thyroxine metabolism with the human microbiome may explain why we observed a significant shift in beta diversity associated with changes in these 2 hormones. Overall, more studies are warranted to understand this relationship.

It should be noted that some significant associations in this study were found for the observed number of ASVs, but not with other alpha-diversity metrics. These additional metrics usually consider other aspects. Specifically, Faith's Phylogenetic Diversity takes into account the evolutionary relatedness of microbial taxa in a community [47]. The Simpson index and Shannon-Weiner Index are both measures of diversity that consider both the richness and evenness of microbial taxa in a community [48, 49]. Therefore, these nonsignificant associations may be explained by the fact that the thyroid dysfunction impacts the richness of microorganisms in the oral environment, but not other aspects, such as evenness and evolutionary relatedness.

The strengths of our study should be considered in light of its limitations. First, NHANES is cross-sectional, thus we cannot examine the temporality between thyroid function and the oral microbiome diversity. It is unclear whether thyroid dysfunction leads to changes in the oral microbiome, or vice versa, or the relationship is reciprocal. However, as the first large-scale study to link the oral microbiome with thyroid function, findings from this study still provide insights for future directions. Second, the composition of the human oral microbiome can be influenced by various factors, including the intake of food and beverages, the availability of endogenous nutrients, the host immune system, medication treatments, and systemic diseases. However, despite these potential influences, the oral microbiome exhibits significant resilience, particularly when compared to the microbial community in the large intestine. Apart from excessive and frequent consumption of fermentable carbohydrates or supplementation with nitrates, the diet has minimal impact on the composition of the oral microbiome [50]. Third, the unweighted number of participants with hyperthyroidism and hypothyroidism is actually low in this study. In the unweighted population, only 13 and 14 participants had clinical hypothyroidism and hyperthyroidism, respectively. Influence of random error cannot be ruled out given the small sample size. However, the NHANES sample design and weighting strategy allow us to produce estimates representative of the US general population. Our results also demonstrated sufficient statistical power. Fourth, some confounders, such as radiotherapy, that may have direct effects on oral microbiome diversity and thyroid functions, are unmeasured in NHANES.

Overall, this study is a large-scale study to investigate the association between the oral microbiome and thyroid function and fill the knowledge gap in this area. In this study, hyperthyroidism was associated with reduced oral microbiome diversity, while high TPOAb levels were associated with increased oral microbiome diversity. Some microbiome compositional differences were observed between FT3 and FT4 groups. All these findings advance our understandings of the role of the oral microbiome in human health and its link with thyroid function, providing insights for future research.

## Data Availability

The data underlying this article are publicly available on the National Health and Nutrition Examination Survey Homepage (cdc.gov), accessed via the link https://www.cdc.gov/nchs/nhanes/index.htm.

## Abbreviations

AL: attachment loss
ASV: amplicon sequence variant
FT3: triiodothyronine
free FT4: free thyroxine
NHANES: US National Health and Nutrition Examination Survey
NMDS: non-metric multidimensional scaling
PD: probing depth
TG: thyroglobulin
TGAb: thyroglobulin antibodies
TT3: total triiodothyronine
TT4: total thyroxine
TPOAb: thyroid peroxidase antibodies
TSH: thyrotropin (thyroid stimulating hormone)
